# Halloysite Nanotubes Supported Ag and ZnO Nanoparticles with Synergistically Enhanced Antibacterial Activity

**DOI:** 10.1186/s11671-017-1859-5

**Published:** 2017-02-20

**Authors:** Zhan Shu, Yi Zhang, Qian Yang, Huaming Yang

**Affiliations:** 1 Centre for Mineral Materials, School of Minerals Processing and Bioengineering, Central South University, Changsha, 410083 China; 2 Hunan Key Laboratory of Mineral Materials and Application, Central South University, Changsha, 410083 China; 3 UCL Cancer Institute, University College London, London, WC1E 6DD UK; 4 State Key Laboratory of Powder Metallurgy, Central South University, Changsha, 410083 China

**Keywords:** Halloysite nanotubes, Nanocomposites, Ag nanoparticles, ZnO nanoparticles, Antibacterial activity

## Abstract

Novel antimicrobial nanocomposite incorporating halloysite nanotubes (HNTs) and silver (Ag) into zinc oxide (ZnO) nanoparticles is prepared by integrating HNTs and decorating Ag nanoparticles. ZnO nanoparticles (ZnO NPs) and Ag nanoparticles (Ag NPs) with a size of about 100 and 8 nm, respectively, are dispersively anchored onto HNTs. The synergistic effects of ZnO NPs, Ag NPs, and HNTs led to the superior antibacterial activity of the Ag-ZnO/HNTs antibacterial nanocomposites. HNTs facilitated the dispersion and stability of ZnO NPs and brought them in close contact with bacteria, while Ag NPs could promote the separation of photogenerated electron-hole pairs and enhanced the antibacterial activity of ZnO NPs. The close contact with cell membrane enabled the nanoparticles to produce the increased concentration of reactive oxygen species and the metal ions to permeate into the cytoplasm, thus induced quick death of bacteria, indicating that Ag-ZnO/HNTs antibacterial nanocomposite is a promising candidate in the antibacterial fields.

## Background

Antibacterial materials such as metals [1–3] and metal oxides [4] inhibit bacteria growth by oxidative stress with the production of reactive oxygen species. Zinc oxide (ZnO) is one of representative metal oxide semiconductors used as commercially antibacterial materials due to low-cost, abundance, and environmentally friendly feature. Several studies have proposed the antibacterial mechanism of zinc oxide nanoparticles (ZnO NPs) to be damaging the cell membrane and releasing reactive oxygen species [5–8]. However, the easy aggregation into big cluster of ZnO NPs at nanoscale in the solution will weaken the antibacterial effect [5]. The low-photoinactivation efficiency in visible region also impose a negative influence on their antibacterial activity.

The dispersibility of ZnO NPs in aqua can be improved by surface modification, but the highly expensive surfactant increases the manufacture cost, including polyvinylpyrrolidone (PVP), oleic acid (OA), together with diethanolamine (DEA), polyethylene glycol methyl ether (PGME), poly(methyl methacrylate) (PMMA), and polystyrene (PS). Also, graphite sheet and carbon nanotubes possessed larger specific surface area, which can indeed facilitate the dispersion of nanoparticles, but their easy carbonization at high-temperature, high-cost, and complicated preparation process will limit their large-scale applications, whereas halloysite nanotubes (HNTs) as the support could make up for the above disadvantages to some extent. Natural clay minerals, such as kaolinite [9, 10], halloysite [11], montmorillonite [2, 12–16], and palygorskite [17–20] are widely used in the catalysis, energy storage, and wastewater treatment application by loading the traditional nanomaterials, which means that they can be used as cost-efficient matrix to improve the dispersion of ZnO given to their natural nanostructures, unique ion exchange capacities, superior hydrophily, and excellent mechanical properties. Such features may not only bring ZnO NPs to be closer to the membrane of bacteria to hamper the normal function of bacteria [21] but also increase the local zinc concentration to inhibit the growth of bacteria [22]. A series of novel metal nanoparticles such as gold [3], silver [23], and copper [24] have strong bactericidal activities for bacteria, fungi, and virus. Using a combination of noble metal and metal oxide antibacterial agent, bacterial growth and survival is believed to be effectively inhibited.

Halloysite (i.e., halloysite nanotubes, HNTs) as a dioctahedral 1:1 nanoclay of the kaolin group, consists of hollow cylinders formed by multiple rolled layers [25–29]. Halloysite-based nanocomposites have gained specific research attention as a potential material for various biological applications (e.g., antibacterial, enzyme immobilization, and controlled drug delivery) [30]. Such interest can be hugely attributed to their physicochemical properties: tubular structures, high-specific surface area, length-to-diameter (L/D) ratio [31, 32], and hydrophobicity. Ag nanoparticles (Ag NPs), as one of the most commercialized bactericidal materials, exhibit higher toxicity to microorganisms by penetrating through the membrane and inducing cell death [1, 15, 33]. Halloysite facilitates the dispersity and controls the distribution of ZnO NPs and brought them close to Ag NPs within 1–10 nm. In this way, ZnO NPs and Ag NPs could efficiently have contact with bacteria cell membrane and remarkably interrupt the membrane functions. A small amount of loaded Ag NPs can achieve the synergistic antimicrobial effect, which could cause direct damage to the bacterial cell membrane [11] and dramatically enhance the antibacterial activity of ZnO NPs. In this paper, Ag-ZnO/HNTs antibacterial nanocomposites were prepared by incorporating HNTs and Ag NPs into ZnO NPs. The interfacial characteristics of ZnO NPs, Ag NPs, and HNTs were investigated. A typical bacterium *Escherichia coli* was used to assess the antibacterial activity of Ag-ZnO/HNTs antibacterial nanocomposites and enhanced antibacterial mechanism was proposed.

## Methods

Raw halloysite mineral was obtained from Chenxi, Hunan province in China. The visible impurities like the brown and black parts were eliminated through hand-selecting process, the white halloysite mineral was milled in an agate mortar before all of the powders passed a 300 mesh sieve. The powder was immersed in water and magnetically stirred for 2 h, then filtered and washed by ethanol, followed by drying at 60 °C for 2 h, finally for the experiment use. A typical process for the synthesis of ZnO/HNTs nanocomposites is described as follows: 2.4 g HNTs, 3.2 g CO(NH_2_)_2_, and 3.2 g Zn(NO_3_)_2_∙6H_2_O were dispersed in 50 mL distilled water, ultrasonic dispersion for 15 min and stirred for 3.5 h at 95 °C, and then calcined at 400 °C for 4 h, labeled as ZnO/HNTs. ZnO/HNTs with different ZnO loading (15, 30, 45, and 60%) were prepared by changing the ZnO:HNTs mass ration. For comparison purpose, pure ZnO was synthesized using the same conditions without adding HNTs. As for the synthesis of Ag-ZnO/HNTs nanocomposites, 2 g ZnO/HNTs, 0.07 g AgNO_3_, and 0.1 g PVP were dispersed in 40 ml distilled water under ultrasonic dispersion for 15 min. Ten milliliter aqueous solution contained 0.02 g NaBH_4_ was added dropwise under stirred for 30 min. The products were further washed with ethanol and water for several times, dried under vacuum at room temperature, and labeled as Ag-ZnO/HNTs.

The X-ray diffraction (XRD) measurements were recorded on a DX-2700 X-ray diffractometer using Cu Kα radiation (*λ* = 0.15406 nm). Data were collected from 2θ range of 5–80° with a scan rate of 0.02°/s and at 40 kV and 40 mA. The morphology and the nanostructure of the samples were observed using a JEOL JSM-6360LV scanning electron microscope (SEM) at an accelerating voltage of 5 kV. Transmission electron microscopy (TEM) studies were performed using a JEOL JEM-2100 F operating at 200 kV. The particle size and lattice distance of samples were observed with a high-resolution transmission electron microscope (HRTEM, JEM-3010; JEOL). X-ray photoelectron spectroscopy (XPS) measurements were taken using a spectrometer (ESCALAB 250; Thermo Fisher Scientific).

Gram-negative *Escherichia coli* (*E. coli*) was used to test the antibacterial activity of the samples (ZnO, ZnO/HNTs, and Ag-ZnO/HNTs). Luria Bertani (LB) broth and nutrient agar were used as sources for culturing *E. coli* at 37 °C in aerobiosis on the rotary platform. The bacterial was in series diluted to reach the concentration for plate count method. Ten milligram nanomaterial was resuspended in the test tube contained 10 mL LB liquid, 2 mL *E. coli* was pipetted into the test tubes and placed in a rotary platform at 37 °C for 4 h. To ensure that any decrease in bacterial number was due to the exposure to the nanomaterial treatment, control group was included in the experiment with the absence of nanomaterial. One hundred microliter samples were transferred onto the LB nutrient agar plates and sprayed evenly on top of the plates using a sterile glass rod. After the bacteria were dried, the petri plates were inverted and incubated at 37 °C for 18–20 h, visible colonies were quantified after incubation.

TEM analysis was performed to observe the effect of Ag-ZnO/HNTs on morphology and surface structure of the bacterial cells. TEM images of samples were accomplished using the following procedures: the cells exposed to Ag-ZnO/HNTs for 4 h were centrifuged and fixed with 2.5% glutaraldehyde overnight at 4 °C, followed by washing with 0.1 M PBS, and then postfixed with 1% osmium tetroxide for 1 h, dehydrated in graded concentrations of ethanol, and embedded in epoxy resin. The resin embedded cells was polymerized at 60 °C overnight. Thick 1~2 μm and thin 90 nm sections were cut using an ultramicrotome (LEICA EM UC7). Grids were stained with uranyl acetate and lead citrate stains. Ultrathin 90 nm sections were examined with TEM transmission electron microscope (HT7700) operated at 80 kV.

## Results and Discussion

**Fig. 1:**
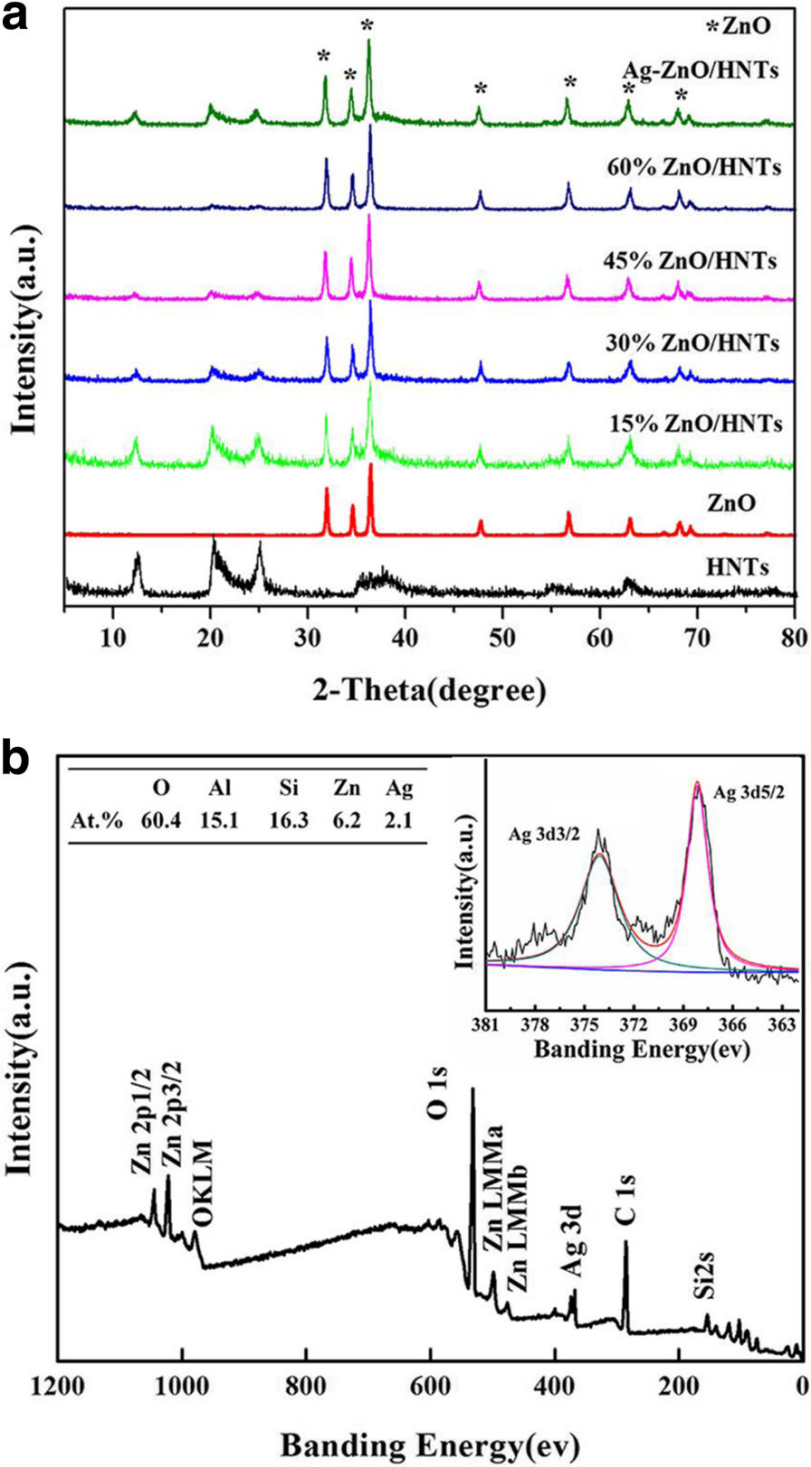
**a** XRD patterns of HNTs, ZnO, ZnO/HNTs with different ZnO loading, and Ag-ZnO/HNTs and **b** XPS spectra of Ag-ZnO/HNTs and atomic concentrations of different elements for Ag-ZnO/HNTs

The above design revealed that the Ag-ZnO/HNTs antibacterial nanocomposites exert an obvious inhibition to *E. coli*. Thus, the features of the simple manufacturing procedure shall be discussed. ZnO showed peaks resembling to that of wurtzite crystallite (JCPDS card no. 36-1451) with characteristics at 31° (*d* = 2.8 Å), 34° (*d* = 2.6 Å), and 36° (*d* = 2.5 Å), which corresponds to the crystallographic orientations of (100), (002), and (101), respectively. The XRD patterns of ZnO/HNTs confirmed that the characteristic data of halloysite (JCPDS card no. 09-0451), which appeared at 11.5° (*d* = 7.58 Å), 20° (*d* = 4.4 Å), and 24.6° (*d* = 3.6 Å) corresponds to the crystallographic orientations of (001), (020), and (002), respectively. The reflections of halloysite became weaker as the amount of ZnO increases (Fig. 1a), while the characteristic data of Ag (JCPDS card no. 04-0783) has not appeared in the reflections due to relative small amount in antibacterial nanocomposites. Full-range XPS spectra of Ag-ZnO/HNTs have been applied to verify the existence of Ag NPs, and Si 2p, Al 2p, O 1 s, Zn 2p, and Ag 3d were detected (Fig. 1b). The Ag 3d spectrum consists of two components (3d5/2 and 3d3/2) which were separated by 6.0 eV. Peaks observed at 368.0 eV (3d5/2 component) and 374.0 eV (3d3/2 component) corresponding to metallic Ag [34] also revealed that the Ag NPs existed in Ag-ZnO/HNTs.

**Fig. 2:**
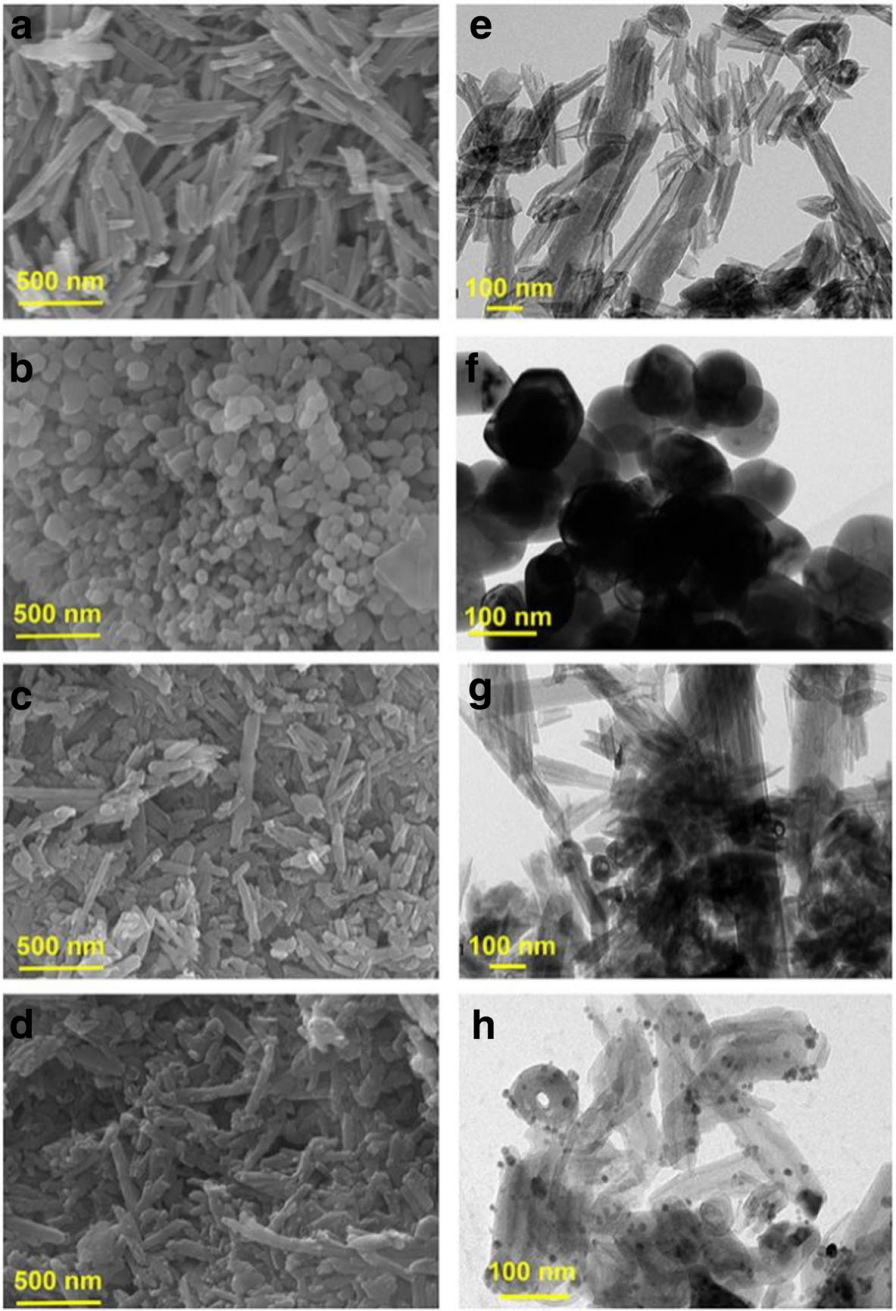
SEM images of **a** HNTs, **b** ZnO, **c** ZnO/HNTs, and **d** Ag-ZnO/HNTs; TEM images of **e** HNTs, **f** ZnO, **g** ZnO/HNTs, and **h** Ag-ZnO/HNTs, respectively

Furthermore, TEM images of HNTs, ZnO, ZnO/HNTs, and Ag-ZnO/HNTs were presented in Fig. 2. Large ZnO NPs with the particle size ranging from 100 to 150 nm were formed by the spontaneous agglomeration of small-sized ZnO NPs. HNTs have been used to minimize the agglomeration of ZnO NPs and facilitate more active sites of ZnO NPs exposed. HNT has shown a short cylindrical hollow tube with an average length of 0.7–1.5 μm, with an external diameter of 50–75 nm, and an internal diameter of 10–30 nm (Fig. 2a, e). After assembling Ag NPs, the characteristic tube morphology of the original HNTs has been retained. Ag NPs (1.31 wt/%) were highly dispersed on the external surfaces of the ZnO/HNTs with particle size of about 8 nm.

**Fig. 3:**
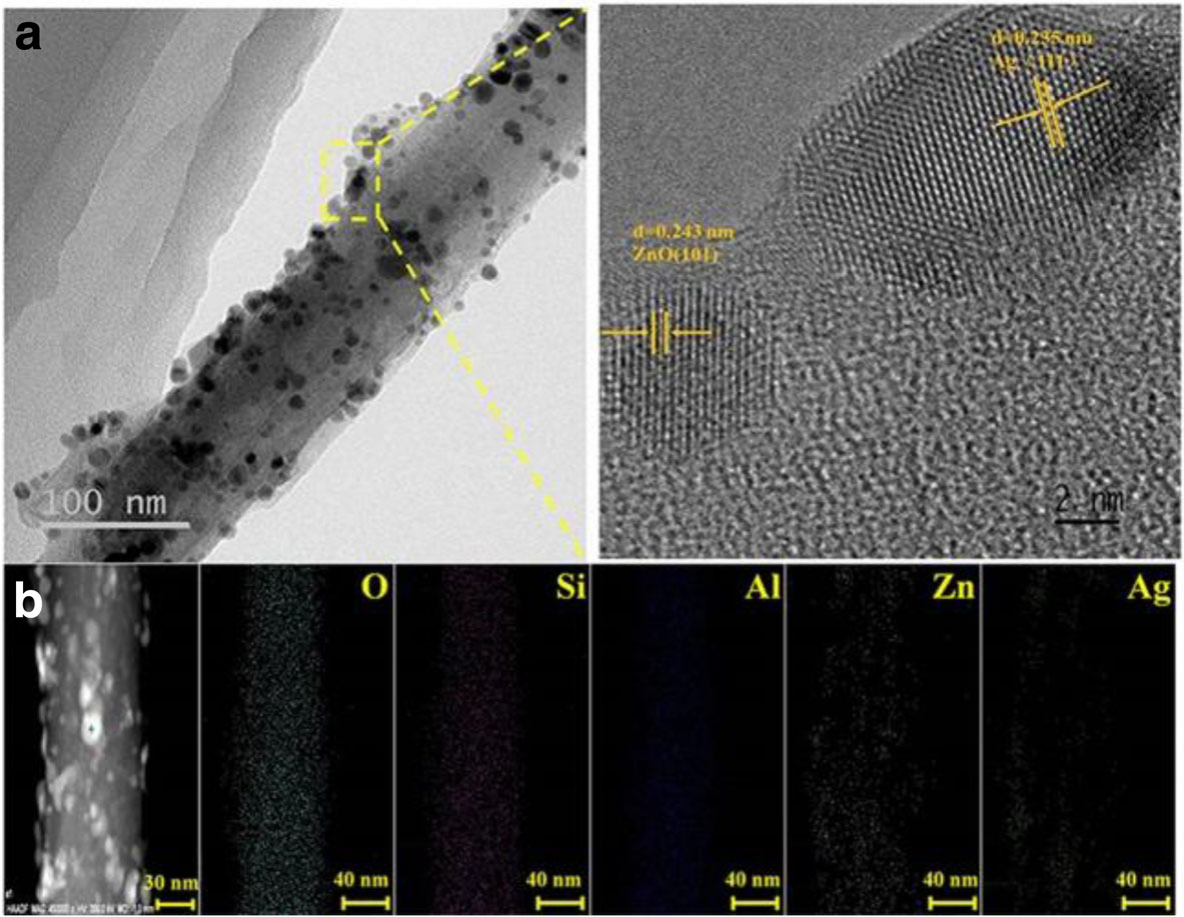
**a** TEM images and the corresponding HRTEM of Ag-ZnO/HNTs and **b** EDS elemental mappings of O, Si, Al, Zn, and Ag elements

More interfacial characteristics of Ag-ZnO/HNTs antibacterial nanocomposites were observed by high-resolution TEM (HRTEM) as shown in Fig. 3. Large ZnO and smaller Ag NPs were densely deposited on the surface of the HNTs, with a size of about 100 and 8 nm, respectively. Energy dispersive X-ray spectroscopy (EDX) elemental mappings of O, Si, Al, Zn, and Ag elements with corresponding TEM image further demonstrated the uniform distribution of Zn and Ag elements in the whole nanocomposites. Signals from Zn and Ag were distributed on the entire tube body consistent with the Si and Al mappings results, validated structure as expected, and indicated that HNTs facilitated the dispersion and stability of ZnO and Ag NPs.

**Fig. 4:**
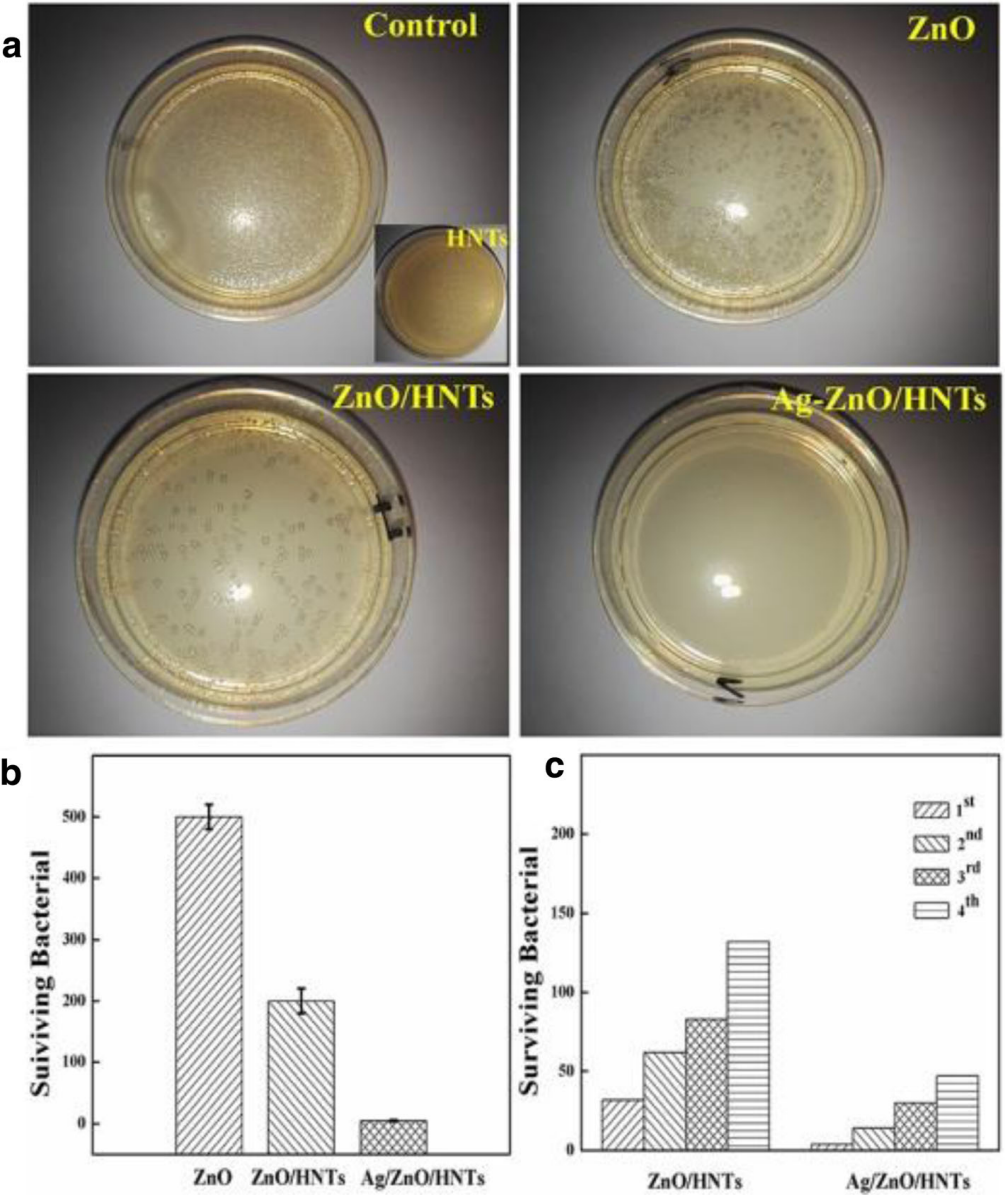
**a** Photographs of the bacterial culture plates, **b** the colony forming units, and **c** antibacterial stability of control, ZnO, HNTs (inset), ZnO/HNTs, and Ag-ZnO/HNTs (1st, 2nd, 3rd, 4th corresponding to the reused time of nanocomposites, respectively)

The antimicrobial activity of the ZnO/HNTs nanocomposites with different ZnO loading was performed to determine the proper mass ratio of ZnO. Thirty percent ZnO loading amount exhibited the best efficacy, which could be explained by the agglomeration of ZnO NPs with excessive ZnO loading (45 and 60%) and the insufficient amount of ZnO antibacterial agents with only 15% ZnO loading. The antimicrobial activity of pure ZnO, HNTs (inset), ZnO/HNTs, Ag-ZnO/HNTs against *E. coli*, and the control group was shown in Fig. 4. The colony forming units (CFU) of both HNTs and the control sample showed normal growth on the agar plates, and the CFU cannot be counted accurately, indicating that pristine HNTs showed no antibacterial activity. The growth inhibition of bacteria was influenced by ZnO, and HNTs showed very low cytotoxic effect. ZnO/HNTs revealed more obvious inhibition on bacteria growth than that on equivalent doses of pure ZnO. Most significantly, Ag-ZnO/HNTs nanocomposites exerted the highest antibacterial activity and stability than that of the equivalent doses of ZnO/HNTs and pure ZnO, which could attribute to the Ag introduced.

**Fig. 5:**
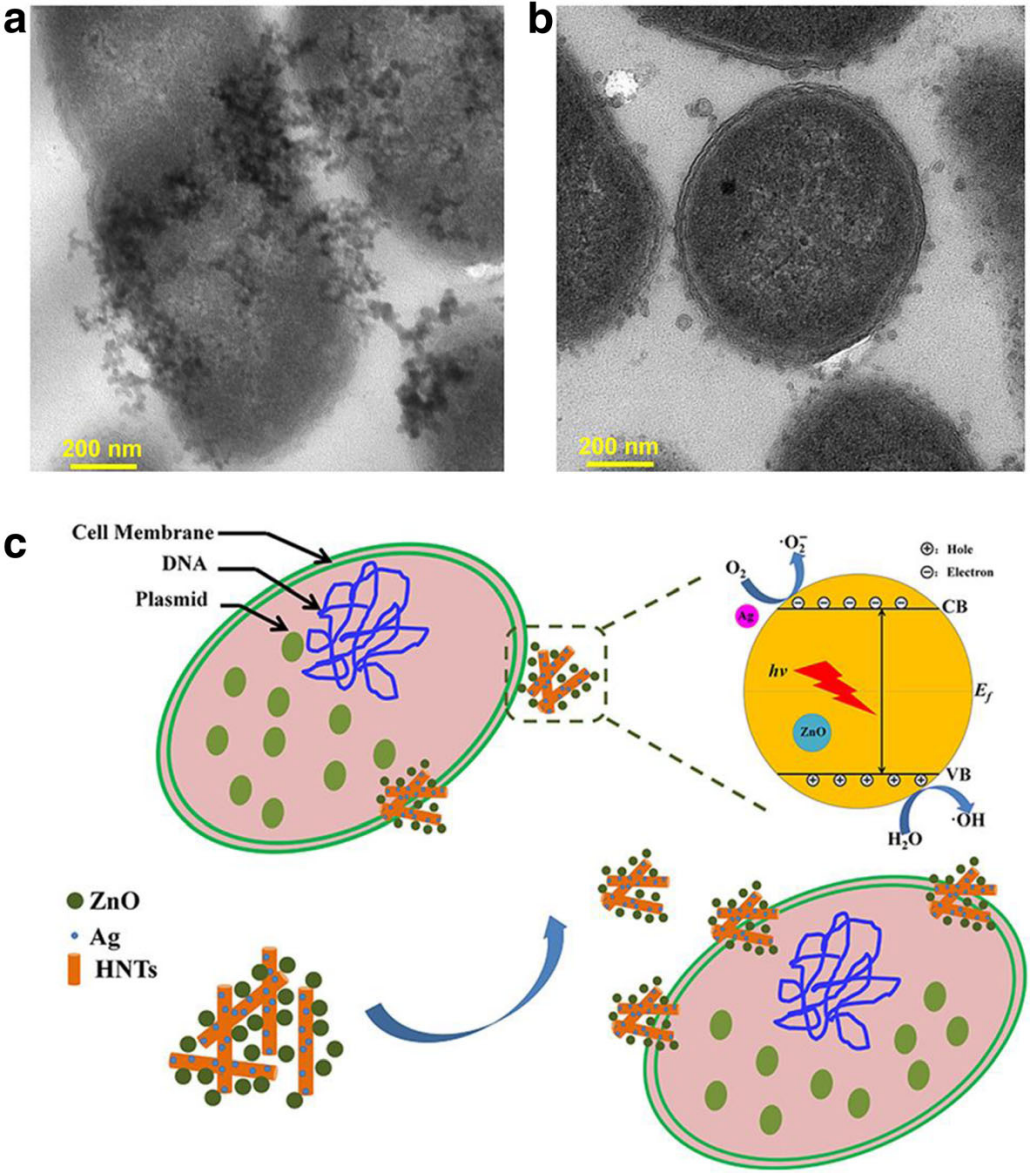
**a**, **b** TEM observations of Ag-ZnO/HNTs nanocomposites absorption in bacteria *E. coli* and **c** schematic diagram for enhanced antibacterial activity

An antibacterial mechanism of Ag-ZnO/HNTs antibacterial nanocomposites against *E. coli* is thus proposed. Bio-TEM has been used to look for any ultrastructural changes as shown in Fig. 5. Large numbers of Ag-ZnO/HNTs antibacterial nanocomposites were detected on bacteria membrane and the plasmid. Ag-ZnO/HNTs antibacterial nanocomposites adsorbed onto the bacterial surface and localized in the periplasmic compartment of bacteria (Fig. 5a, b), the production of ROS played a crucial role in causing its disorganization. Most researchers demonstrated that nanoparticles were well attached to the bacteria membrane and could produce elevated level of reactive oxygen species (ROS), mostly hydroxyl radicals given by the reaction of electrons and H_2_O under visible light. Singlet oxygen generated from the O_2_ by holes, which could oxidize the cell content and cause bacterial disorganization. Among the Ag-ZnO/HNTs antibacterial nanocomposites, halloysite could facilitate the dispersion and stability of ZnO NPs and draw the Ag-ZnO/HNTs nanocomposites in close contact with the bacterial membrane. ZnO NPs with higher dispersion may have increased surface area, causing more active sites to produce more ROS. Ag NPs decorated ZnO NPs promote the separation of photogenerated electron-hole pairs or direct damage to the bacterial cell membrane, which could dramatically enhance the antibacterial activity of ZnO NPs. In the incorporation of the superior antibacterial activities of Ag NPs and excellent dispersibility of halloysite, antibacterial nanocomposites will show a higher antibacterial activity. Above observations are crucial for explaining the antibacterial mode of operated Ag-ZnO/HNTs antibacterial nanocomposites.

## Conclusions

Ag-ZnO/HNTs antibacterial nanocomposites were prepared by incorporating HNTs and Ag NPs into ZnO NPs. HNTs facilitated the dispersion and stability of ZnO NPs and brought them in close contact with bacteria. Ag NPs promote the separation of photogenerated electron-hole pairs and enhance the antibacterial activity of ZnO NPs. ZnO/HNTs shown evident inhibition on bacteria growth with increased nanocomposite concentration than that on equivalent doses of pure ZnO. Ag-ZnO/HNTs nanocomposites showed the highest antibacterial activity and stability. The outstanding results demonstrated excellent antibacterial properties of Ag-ZnO/HNTs antibacterial nanocomposites.

## Abbreviations

Ag NPs: Ag nanoparticles
CFU: Colony forming units
DEA: Diethanolamine
E. coli: *Escherichia coli*
HNTs: Halloysite nanotubes
HRTEM: High-resolution transmission electron microscope
L/D: Length to diameter
OA: Oleic acid
PGME: Polyethylene glycol methyl ether
PMMA: Poly(methyl methacrylate)
PS: Polystyrene
PVP: Polyvinylpyrrolidone
SEM: Scanning electron microscope
TEM: Transmission electron microscopy
XPS: X-ray photoelectron spectroscopy
XRD: X-ray diffraction
ZnO NPs: ZnO nanoparticles
ZnO: Zinc oxide
